# Heterodimeric IL-15 in Cancer Immunotherapy

**DOI:** 10.3390/cancers13040837

**Published:** 2021-02-17

**Authors:** Cristina Bergamaschi, Vasiliki Stravokefalou, Dimitris Stellas, Sevasti Karaliota, Barbara K. Felber, George N. Pavlakis

**Affiliations:** 1 Human Retrovirus Pathogenesis Section, Vaccine Branch, Center for Cancer Research, National Cancer Institute at Frederick, Frederick, MD 21702-1201, USA; cristina.bergamaschi@nih.gov (C.B.); barbara.felber@nih.gov (B.K.F.); 2 Human Retrovirus Section, Vaccine Branch, Center for Cancer Research, National Cancer Institute at Frederick, Frederick, MD 21702-1201, USA; vasiliki.stravokefalou@nih.gov (V.S.); dimitrios.stellas@nih.gov (D.S.); sevasti.karaliota@nih.gov (S.K.); 3 Basic Science Program, Frederick National Laboratory for Cancer Research, Frederick, MD 21702-1201, USA

**Keywords:** cytokine, IL-15, IL-15 receptor alpha (IL-15Rα), heterodimeric IL-15 (hetIL-15), cancer immunotherapy, cytotoxic cells, clinical trials

## Abstract

**Simple Summary:**

The rapidly expanding field of cancer immunotherapy uses diverse technologies, including cytokines, T cells, and antibody administration, with the aim to induce effective immune responses leading to tumor control. Interleukin-15 (IL-15), a cytokine discovered in 1994, supports the homeostasis of cytotoxic immune cells and shows promise as an anti-tumor agent. Many studies have elucidated IL-15 synthesis, regulation and biological function and explored its therapeutic efficacy in preclinical cancer models. *Escherichia coli*-derived single-chain IL-15 was tested in the first in-human trial in cancer patients. Its effects were limited by the biology of IL-15, which in vivo comprises a complex of the IL-15 chain with the IL-15 receptor alpha (IL-15Rα) chain, together forming the IL-15 heterodimer (hetIL-15). Currently, single-chain IL-15 and several heterodimeric IL-15:IL-15Rα variants (hetIL-15, N-803 and RLI) are being tested in clinical trials. This review presents a summary of contemporary preclinical and clinical research on IL-15.

**Abstract:**

Immunotherapy has emerged as a valuable strategy for the treatment of many cancer types. Interleukin-15 (IL-15) promotes the growth and function of cytotoxic CD8^+^ T and natural killer (NK) cells. It also enhances leukocyte trafficking and stimulates tumor-infiltrating lymphocytes expansion and activity. Bioactive IL-15 is produced in the body as a heterodimeric cytokine, comprising the IL-15 and the so-called IL-15 receptor alpha chain that are together termed “heterodimeric IL-15” (hetIL-15). hetIL-15, closely resembling the natural form of the cytokine produced in vivo, and IL-15:IL-15Rα complex variants, such as hetIL-15Fc, N-803 and RLI, are the currently available IL-15 agents. These molecules have showed favorable pharmacokinetics and biological function in vivo in comparison to single-chain recombinant IL-15. Preclinical animal studies have supported their anti-tumor activity, suggesting IL-15 as a general method to convert “cold” tumors into “hot”, by promoting tumor lymphocyte infiltration. In clinical trials, IL-15-based therapies are overall well-tolerated and result in the expansion and activation of NK and memory CD8^+^ T cells. Combinations with other immunotherapies are being investigated to improve the anti-tumor efficacy of IL-15 agents in the clinic.

## 1. Introduction

Interleukin-15 (IL-15) belongs to the γ-chain family of cytokines, that includes also IL-2, IL-4, IL-7, IL-9 and IL-21. These cytokines bind to the same γ-chain in their receptor complex and have unique and overlapping roles in regulating the development, maintenance, trafficking, and function of different lymphocyte subsets [1,2,3,4,5]. IL-15 is a growth, mobilization, and activation factor for many leukocyte populations, including natural killer (NK) cells, canonical αβTCR^+^ CD8^+^, γδTCR^+^, natural killer-T (NK-T), and intraepithelial T lymphocytes [1,6,7,8]. The non-redundant role of IL-15 in stimulating the cytotoxic activity of immune cells has supported the clinical development of IL-15 for cancer immunotherapy.

IL-15 was discovered as a molecule highly related to interleukin-2 (IL-2), which reached the clinical setting as an approved biological drug in 1992 and was the first paradigm of successful immunotherapy of cancer [9,10]. IL-15 and IL-2 share the same IL-2 receptor β/γ dimer [11], but their systemic biological effects are different, as shown by studies performed using knockout (KO) mice. Lack of IL-2 causes immune activation and severe autoimmunity [12,13,14,15], whereas lack of IL-15 results in almost complete absence of NK cells and a severe reduction in peripheral CD8^+^ T lymphocytes [16]. The biological differences between IL-2 and IL-15 are determined by their different production sites [1,2,17,18], their strength of association with the specific membrane binding proteins IL-2 receptor alpha (IL-2Rα) and IL-15 receptor alpha (IL-15Rα) [19], respectively, and the regulation of these molecules. IL-15 is not produced by lymphocytes; instead, it is produced from myeloid cells, stroma cells of many organs, and blood endothelial cells. Unlike IL-2Rα, the specific IL-15 binding protein named IL-15Rα does not serve a receptor function, but it has evolved to be the other half of the IL-15 cytokine. Indeed, the cytokine is produced and functions as a heterodimer of two polypeptide chains, IL-15 and IL-15Rα [20], named heterodimeric IL-15 (hetIL-15). hetIL-15 has shown anticancer activity in many model systems and is presently in multiple clinical trials for cancer immunotherapy.

In this review, we present the latest knowledge about hetIL-15 synthesis and regulation, about the mechanisms of function of this cytokine on the immune system, and an overview of the clinical applications of hetIL-15 in cancer immunotherapy. A comprehensive understanding of the unique biology of the IL-15 heterodimeric cytokine will allow for more rational design of IL-15-related studies at all levels, from basic biology to clinical research.

## 2. Regulation of Heterodimeric IL-15 Production: Trans-Presentation and Soluble hetIL-15

IL-15 was identified by two independent groups in 1994 as a 14 to 15 kDa T cell growth factor similar to IL-2 in the simian kidney epithelial cell line [21], and in the human T cell leukemia virus-1 cell line [22]. Nucleotide or protein comparison failed to show any similarities between IL-15 and IL-2, but crystal structure analysis revealed that IL-15 has a four helix “up-up-down-down” structure [23], like IL-2 and other member of the same superfamily of cytokines. IL-15 is conserved among species, and human IL-15 shows 96% sequence homology with simian IL-15 [21,24] and 72% with mouse [25] and rat IL-15.

Production of IL-15 is tightly regulated. In addition to regulation at the transcriptional level, IL-15 expression is controlled at several post-transcriptional and post-translational steps, such as mRNA stability, the generation of alternative spliced isoforms, intracellular trafficking, and secretion [26,27].

**Figure 1:**
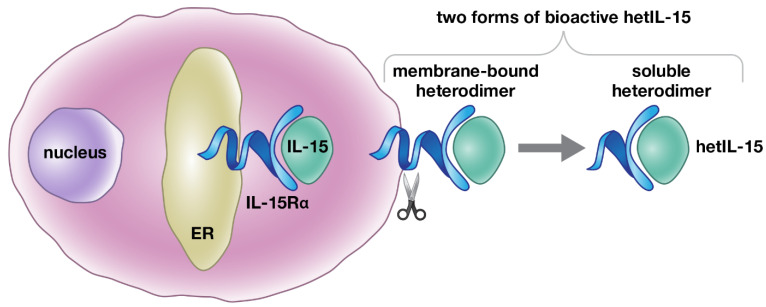
Schematic representation of hetIL-15 production. IL-15 is a heterodimeric cytokine, comprising the IL-15 and IL-15Rα chains that are together termed heterodimeric IL-15 (hetIL-15). Simultaneous expression of IL-15Rα in the same cells (dendritic cells, monocyte/macrophages, and others) is necessary for the production and secretion of IL-15 under physiological conditions. Upon co-expression, the two chains, IL-15 and IL-15Rα, associate in the endoplasmic reticulum (ER), due to their high binding affinity (K_d_ = 10^−11^ M). The IL-15 heterodimer is then transported to the cell surface and released as a bioactive soluble heterodimeric molecule, upon proteolytic cleavage of IL-15Rα.

IL-15 has been reported to have a unique mechanism of action in vivo among the common γ-chain cytokines. IL-15 signals through the common IL-2/IL-15 receptor β/γ complex [11]. Both IL-2 and IL-15 use additional unique and evolutionarily related surface binding proteins responsible for the specificity of binding, which were named IL-2Rα and IL-15Rα, respectively. IL-2Rα displays a lower affinity for IL-2 (K_d_~10^−8^ M) in the absence of IL-2 receptor β/γ, and acts by becoming part of the receptor. IL-15Rα has a high affinity for IL-15 (K_d_~10^−11^ M) [19]. Dubois et al. [28] showed that IL-15 acts on the surface of producing cells in complex with the membrane-anchored IL-15Rα to engage the IL-2/IL-15 receptor β/γ complex in nearby cells, a process termed trans-presentation. Furthermore, in physiological conditions, IL-15 and IL-15Rα expression from the same cells is required in order to achieve biological activity; bone marrow from chimeric mice repopulated with a mixture of IL-15-/- and IL-15Rα-/- cells failed to generate memory CD8^+^ T cells and mature NK cells [29,30,31]. An additional observation was that IL-15 and IL-15Rα genes present similarities in their promoter function and are co-transcribed in different cell types [19,28,32,33,34]. The molecular mechanism explaining these intriguing observations has been reported by our group and others [35,36]. At the cellular level, co-expressed IL-15 and IL-15Rα rapidly associate in the endoplasmic reticulum (ER) to form a stable heterodimeric membrane-bound complex that is exported to the cell membrane, where it stimulates adjacent IL-15Rβ/γ-expressing cells [35,37]. In the absence of IL-15Rα, IL-15 is highly unstable and is rapidly degraded before secretion. On the cell membrane, the complex is released as a bioactive soluble heterodimeric cytokine upon proteolytic cleavage of IL-15Rα (Figure 1). Indeed, the heterodimer of the IL-15 chain with the IL-15Rα chain was found circulating in the plasma of mice and humans [20]. Overall, IL-15Rα acts as a chaperone molecule, influencing the bioavailability of IL-15 and expanding IL-15 effects from autocrine or juxtacrine to paracrine or endocrine modes [35,36]. Therefore, IL-15Rα is part of a heterodimeric IL-15 cytokine, rather than functioning as a cytokine receptor [35]. These results are in agreement with the reported ability of recombinant soluble IL-15Rα to act as a potent agonist of IL-15 function in vivo [38,39], and support our conclusion that the functional cytokine in vivo is the heterodimer, named hetIL-15.

## 3. Source and Physiological Function of hetIL-15

hetIL-15 stimulates NK, NK-T, γδ T, type-1 innate lymphoid cells (ILC1), memory and tissue resident CD8^+^ T cells [28,29,30,36,40,41,42,43,44,45], while having no strong effects on Tregs and inhibiting activation-induced Cell death (AICD).

IL-15 and IL-15Rα mRNAs are widely and coordinately produced by several cell types, including monocytes, macrophages, dendritic cells, stroma cells from bone marrow and lymph nodes, blood endothelial and intestinal epithelial cells [46,47,48]. IL-15 reporter mouse models confirmed dendritic cells (DCs) and monocytes/macrophages as the primary source of IL-15 [17,18]. GFP reporter expression was found in CD8^+^ DCs in spleen and in both CD8^+^ and CD8^−^ DCs in lymph nodes. Expression of both IL-15 and IL-15Rα can also be upregulated by several signals, including lipopolysaccharide (LPS), type-I interferons, and pathogen-associated molecular patterns (PAMPs), such as double-stranded RNA. In contrast to the widespread expression of IL-15 and IL-15Rα, the cytokine receptor β and γ subunits are more selectively expressed by lymphocytes, with the highest level found on activated NK and CD8^+^ T cells.

Trans-presentation of IL-15 and shedding of soluble hetIL-15 directly stimulates proliferation and survival in IL-15 responsive cells and regulates their biological functions [28]. The IL-15 trans-presentation mechanism applies to CD8^+^, γδ T cells and NK cells [28,29,30,36,40,41,42,43,44,45,49]. In responsive cells, hetIL-15 signaling results in the activation of JAK1 and JAK3 kinases that promote the phosphorylation and dimerization of signal transducer and activator of transcription protein-3 and 5 (STAT3/5). STAT dimers traffic to the nucleus to function as transcriptional factors. Additional pathways activated by hetIL-15 include the PI3K/Akt/mTOR and the RAS/RAF MAPK pathways. Overall, these signaling pathways enhance the expression of genes involved in proliferation and anti-apoptotic signals such as *Bcl-2*, *c-Myc*, *c-Fos*, *c-Jun*, *Mcl-1*, *Bcl-xl*, in aerobic glycolysis, and in effector and cytotoxic immune cell functions and migration, such as *IFN-γ*, *TNF-α*, *XCL1*, *Granzyme* and *Perforin*. hetIL-15 effects on the immune system and the selectivity of hetIL-15 responsiveness makes this cytokine attractive for cancer immunotherapy.

## 4. IL-15:IL-15Rα Complexes as Cytokine Agonists in Animal Models and Their Efficacy in Preclinical Cancer Studies

Immunotherapy is a promising intervention against cancer. Optimized immunotherapeutic strategies need to be implemented to convert the inefficient immune responses commonly found in the tumor environment to effective responses leading to prolonged tumor control. The presence of tumor-infiltrating T cells (TILs) is considered to be one of the most important biomarkers to predict the clinical benefit in response to immunotherapies [50,51,52]. Several studies highlighted the potential anti-cancer efficacy of IL-15-based therapies.

**Figure 2:**
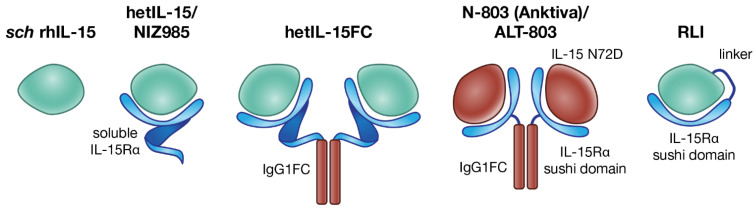
Different IL-15 preparations used in cancer immunotherapy. The single-chain *E. coli*-derived IL-15 (*sch* rhIL-15, left) [53] is the first IL-15 tested in the clinic, but its use is limited by the short half-life and potential toxicity. The natural form of IL-15 produced physiologically is the heterodimer of IL-15 with IL-15Rα. Several heterodimeric forms have been developed. From left to right, human embryonic kidney 293 (HEK293)-derived or Chinese hamster ovary (CHO)-derived hetIL-15/NIZ985, the soluble heterodimer of IL-15 with IL-15Rα, upon natural shedding of IL-15Rα from the membrane of producing cells [54]; human HEK293-derived hetIL-15FC, a dimeric form in which soluble IL-15Rα is fused to the Fc region of IgG1; N-803, also known as Anktiva and ALT-803, consisting of a CHO-derived soluble complex comprising mutated IL-15 (IL-15 N72D) and a dimeric IL-15Rα sushi domain-IgG1 Fc fusion protein [55]; Receptor-Linker-IL-15 (RLI) consists of the IL-15Rα sushi domain fused to IL-15, via a 20-amino acid flexible linker [56].

Single chain recombinant human IL-15 (*sch* rhIL-15), hetIL-15, and several IL-15:IL-15Rα fusion proteins (Figure 2) have been produced and tested in preclinical models (mice and monkeys) to determine the pharmacokinetics, the bioavailability and the bioactivity of these agents, in order to advance to clinical trials for cancer immunotherapy. *sch* rhIL-15, hetIL-15, and IL-15:IL-15Rα fusion agonists were also all shown to have anti-cancer activity in several mouse tumor models.

### 4.1. sch rhIL-15

*sch* rhIL-15 is a non-glycosylated monomer of ~12 kDa produced in *E. coli* [53]. It has been tested in preclinical studies upon intravenous (IV) [57], subcutaneous (SC) [58], and continuous intravenous infusion (CIV) [58]. *sch* rhIL-15 administration in macaques increased the frequency of cycling peripheral NK and effector memory CD8^+^ T cells [57], as well as promoting cell growth and tissue migration of CD4^+^ effector memory T cells [59]. Although *sch* rhIL-15 treatment was overall well-tolerated, some toxicity was observed, including hypotension, fever, chills, and rigors. At higher doses, a transient drop in circulating neutrophils was observed, alongside the accumulation of granulocytes in the liver [60].

Several studies in mice have demonstrated that *sch* rhIL-15 monotherapy results in tumor growth control, decreased metastatic burden, and increased survival in preclinical models of melanoma, colon and prostate carcinoma and lymphoma. The anti-cancer effect of *sch* rhIL-15 depended on the systemic activation of NK and CD8^+^ T cells and the maintenance of specific anti-tumor T cells [61,62,63,64,65,66,67].

### 4.2. hetIL-15 (NIZ985)

IL-15 is generated as a heterodimeric complex of IL-15 and IL-15Rα molecules in both physiologic and pathologic conditions [20]. Our group has previously reported a systematic approach to reproduce all the natural steps of production and processing of hetIL-15 in engineered human cells. We generated stable, clonal HEK293-derived human cell lines that secrete high levels of glycosylated hetIL-15 cytokine [54,68]. The human hetIL-15 has been licensed by Novartis, and the clinical trials continue under the name NIZ985. Upon administration in mice, hetIL-15 was characterized by an extended half-life and promoted a robust expansion of NK and T cells, demonstrating pharmacokinetics and in vivo bioactivity superior to *sch* rhIL-15 [35,54]. Pharmacokinetics, pharmacodynamic and toxicity profile of hetIL-15 were also evaluated in macaques [69]. hetIL-15 was provided via SC route at the dose of 0.5, 5 and 50 μg/kg, three times/week for a two-week cycle, and resulted in persistent bioactive levels of plasma IL-15 with T_1/2_ of ~12 h. Effects of the treatment included increased blood lymphocyte cell count and cytotoxic cells, proliferation of T cells, and elevated plasma IL-18 levels [69,70]. At the higher 50 μg/kg dose, edema was observed in various organs, including the lower abdomen, extremities, and genital area, and swelling was observed in lymph nodes, along with signs of capillary leak syndrome and kidney dysfunction. Body temperature was generally increased following dose administration [69]. The favorable properties of hetIL-15 allowed the development of delivery protocols that maximized effects while avoiding cytokine spikes, via SC delivery of increasing (doubling) doses of hetIL-15, ranging from 2 to 64 μg/kg in a two-week cycle [69]. Each hetIL-15 dose induces lymphocyte proliferation, which leads to more targets, requiring increasing levels of cytokine for efficient stimulation of the increased lymphocytes. Delivery of hetIL-15 following a doubling step–dose regimen provides sufficient cytokine to sustain the continuous expansion and activation of lymphocytes minimizing any side effects.

**Figure 3:**
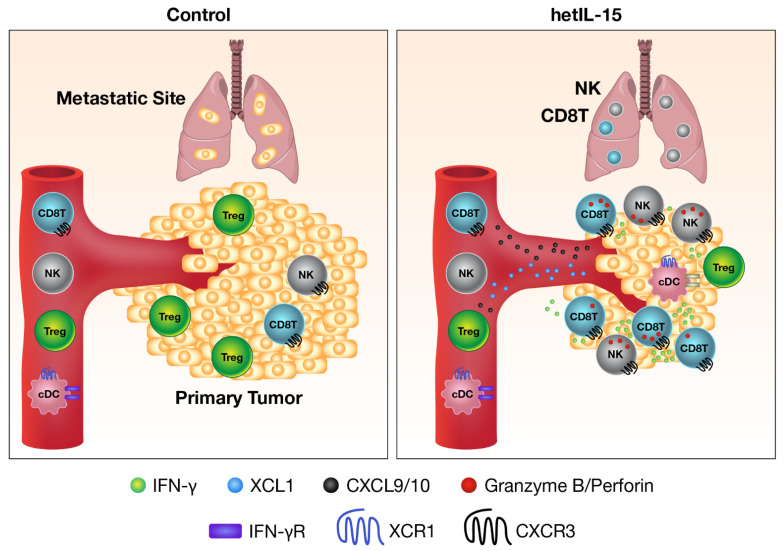
Mechanism of anti-tumor activity of heterodimeric IL-15. hetIL-15 therapy is beneficial against both primary tumor and metastatic disease. Use of hetIL-15 is a general method to push effector lymphocytes inside the tumor. IL-15 directly stimulates the cytotoxicity (granzyme B, red dots) and IFN-γ production (green dots) of CD8^+^ T and NK cells in the tumor microenvironment. IL-15 also induces the secretion of XCL1 (blue dots) from activated lymphocytes, a chemokine responsible for the recruitment of XCR1^+^ conventional dendritic cells (cDC) with cross-priming ability. cDC within the tumor support CD8^+^ T cells effector function and secretes Chemokine (C-X-C motif) ligand 9 (CXCL9) and Chemokine (C-X-C motif) ligand 10 (CXCL10) (black dots) in response to IFN-γ. CXCL9 and CXCL10 create a gradient that attracts more CXCR3^+^ effector CD8^+^ T and NK cells. As a result, the number of CD8^+^ T and NK cells, as well as the CD8/Treg ratio, increase in hetIL-15-treated tumors, leading to tumor growth control.

hetIL-15 therapy has been shown to be effective against both primary tumor and metastatic disease in the B16 melanoma, MC38 colon carcinoma, TC-1 carcinoma, breast, and pancreatic tumor mouse models [71,72] (also shown in our unpublished data). The mechanism of action involves potentiation of multiple pathways, including leukocyte expansion and trafficking to the tumor, increase in cell–cell interactions, anti-tumor cytokine production, and direct cytotoxicity. In the tumor, hetIL-15-stimulated NK, CD8^+^ and CD4^+^ T cells show increased proliferation, survival, and cytotoxic commitment, with high levels of IFN-γ and granzyme B secretion. hetIL-15 treatment causes activated lymphocytes to produce the Chemokine (C motif) ligand 1 (XCL1) that recruits conventional type-1 dendritic cells (cDC1) to the tumor. These cDC1 cells in turn secrete Chemokine (C-X-C motif) ligand 9 (CXCL9) and Chemokine (C-X-C motif) ligand 10 (CXCL10), two IFN-γ-driven chemokines, leading to the recruitment of CXCR3^+^ effector NK and CD8^+^ T cells, that may further produce IFN-γ and generate anti-tumor inflammatory responses in a self-amplifying positive feedback loop (Figure 3) [71]. These data are in agreement with one study demonstrating the role of endogenous IL-15 in inducing higher densities of immune cells at the centers and invasive margins of tumors that correlate with better disease outcomes and increased survival in patients with colorectal cancers [73].

### 4.3. hetIL-15Fc

An additional form of fully glycosylated hetIL-15 in which the C-terminus of soluble IL-15Rα is fused to the Fc region of human IgG1 has been generated and named hetIL-15Fc. In mouse models, hetIL-15Fc was shown to be superior to *sch* rhIL-15 and to possess anti-tumor activity by several investigators [38,39,74,75,76,77]. hetIL-15Fc was characterized by an increased serum half-life and induced a strong expansion of both NK and memory CD8^+^ T cells that was 50-fold more potent than *sch* rhIL-15. The fusion to the Fc region may provide the ability to act as a cell-associated cytokine, mimicking the IL-15 trans-presentation, upon in vivo delivery of the cytokine.

### 4.4. N-803 (Anktiva, Formerly ALT-803)

N-803 consists of a novel IL-15 mutant (N72D) bound to the extracellular region of IL-15Rα fused to IgG1 Fc. It was suggested that the N72D mutation in IL-15 confers a five-fold increase in its biological activity as compared to single-chain wild type IL-15, by enhancing binding to CD122 and CD122/CD132 activation [78]. It is not clear what is the role of N72D mutation on the biological activity of the heterodimeric form. This part of the molecule is prone to deamidation, and it was suggested that production as a heterodimer and N79-glycosylation partially prevents N77-deamidation [68]. The safety, pharmacokinetics, and immunological effects of N-803 have been assessed both in mice [55,79,80,81] and in cynomolgus monkeys [81]. Upon delivery in mice, N-803 stimulated CD11b^+^CD27^hi^ NK cells and memory CD122^+^CD44^+^ T cells and exhibited a 35-fold longer serum half-life than *sch* rhIL-15 [79,81]. Cynomolgus monkeys showed a dose-dependent increase in blood lymphocytes along with mild multifocal lymphocytic infiltration in multiple organs after weekly IV administration of N-803 at 30 and 100 µg/Kg. Administration of N-803 was not accompanied by increased plasma level of inflammatory cytokines IFN-γ, TNF-α, IL-6, IL-5, IL-4, or IL-2. Pharmacokinetic analysis estimated the T_1/2_ of N-803 to be 7.2–8 h. Overall, N-803 showed a wide therapeutic window with no toxicity in vivo [81].

N-803 treatment proved effective in reducing tumor growth and/or in improving survival in several mouse cancer models, including multiple myeloma [82], breast cancer [79], melanoma [79,81], glioblastoma [83], colon carcinoma [79,81], ovarian cancer [84] and lymphoma [85]. As an anti-cancer agent, N-803 promoted the migration of CD8^+^ T and NK cells within the tumor bed and sustained their cytotoxicity and ability to secrete IFN-γ.

### 4.5. RLI (Receptor-Linker-IL-15) Superagonist

The N-terminus region (1-66 aa) of the mature IL-15Rα, called the sushi domain, contains all the structural amino acids responsible for IL-15 binding at high affinity [23]. Additionally, the IL-15Rα sushi domain alone also functions as a potent agonist of IL-15 action [56]. The protein called Receptor-Linker-IL-15 (RLI) consists of the IL-15Rα sushi domain fused to IL-15, via a 20-amino acid flexible linker, and it was shown to act as an IL-15 agonist in vivo. Delivery of RLI to mice resulted in prolonged plasma IL-15 levels and bioactivity similar to hetIL-15Fc complexes [56]. RLI also greatly enhanced the reconstitution of human NK and CD8^+^ T cells in humanized mice, via the strong upregulation of genes involved in cellular activation and survival: *CD69*, *Bcl-2* and *Bcl-xl* [43].

RLI demonstrated strong anti-tumor effects and improved survival in the B16 melanoma model. RLI was shown to reduce primary tumor growth and metastatic burden of human colon carcinoma in an orthotopic nude mouse, through the stimulation of NK cells in absence of T cells [86]. Anti-metastatic effects were also observed in the 4T1 breast cancer model [87].

### 4.6. Optimized DNAs Expressing hetIL-15

The induction of bioactive systemic levels of hetIL-15 has been greatly facilitated by improvements in DNA vector design and delivery methods.

Intramuscular administration of such DNAs delivered by in vivo electroporation resulted in systemic levels of bioactive IL-15 in macaques, accompanied by expansion of NK, γδ TCR^+^ and memory CD8^+^ T cells [88]. This study demonstrated that the injection of hetIL-15-expressing DNAs or hetIL-15 purified protein produced similar results. Muscles are a physiologic location for IL-15 production [89,90,91], and the repeated transient expression of hetIL-15 did not cause any adverse effects in macaques.

Overall, the anti-tumor efficacy of IL-15-based therapies depends on changes in the cellular and cytokine landscape within the tumor. IL-15 is proposed as a general method to enhance leukocyte (such as T, NK cells and cross-priming DCs) entry into tumors, as well as their function, increasing the success rate of immunotherapeutic interventions.

For clinical applications, the advantages of the IL-15:IL-15Rα heterodimeric forms in comparison to *sch* rhIL-15 are: (i) easier production and higher yield of glycosylated forms; (ii) increased stability in vivo; and (iii) increased bioactivity on a molar basis, thereby offering a simpler delivery mechanism and dosing structure. These properties result in a reduced risk of toxicity related to cytokine spikes, making the IL-15:IL-15Rα complex the most favorable form for cancer immunotherapy in humans.

## 5. IL-15 in Combination Therapy for Cancer

The cancer immunotherapy field currently focuses on combining the use of IL-15 with other agents, i.e., checkpoint inhibitors, monoclonal antibodies, chemotherapy, radiation, and chimeric antigen receptor T (CAR-T) cells, in order to target multiple mechanisms and enhance the immune response against tumors. Indeed, the inability of tumor-infiltrating lymphocytes (TILs) to eradicate cancer could be attributed to many factors, such as their functional impairment, secondary to suppression induced by Tregs and other immune-suppression mechanisms, the lack of proper co-stimulation, or Major Histocompatibility Complex (MHC) down-regulation on the target cancer cells, resulting in evasion from immune recognition. The effectiveness of TILs may be limited by a multitude of negative regulatory mechanisms, including anti-inflammatory cytokines (such as IL-10 [92] and TGF-β [93]), inhibitory cells (e.g., Tregs [94] and myeloid-derived suppressor cells [95]), and the up-regulation of inhibitory receptors, such as programmed death-1 (PD-1), and its ligands, programmed death-ligand 1 and 2 (PD-L1 and PD-L2), which have been demonstrated to attenuate immune responses in the context of tumor immunity [96]. Several mechanisms of cancer resistance to therapies are also reviewed in Visconti et al. [97].

### 5.1. Combination of IL-15 with Checkpoint Inhibitors and Monoclonal Antibodies

Several interventions combining IL-15 agents with checkpoint inhibitors, antibodies stimulating immune cell functions (e.g., anti-CD40, anti-CD16), monoclonal antibodies specific for tumor-associated antigens, and immunotoxins have been tested in preclinical models, with promising results.

IL-15 induces the expression of the immune checkpoint molecules PD-1, T-cell Immunoglobulin domain and Mucin domain 3 (TIM-3) and T-cell immunoreceptor with Ig and ITIM domain (TIGIT) on CD8^+^ T cells and promotes the secretion of the anti-inflammatory cytokine IL-10 [65,66]. The combination of *sch* rhIL-15 with both anti-CTLA4 and anti-PD-L1 was tested in the CT26 and MC38 colon carcinoma and in the Transgenic Adenocarcinoma Mouse Prostate C2 (TRAMP-C2) cancer models in mice. Although the single agents only provided limited activity, the triple therapy resulted in significant anti-tumor benefit [65]. Similarly, combination of N-803 with checkpoint inhibitors promoted the development of long-term immune responses, further enhancing the anti-tumor efficacy of both single agents [79,83]. RLI also enhanced the anti-tumor activity of PD-1 antagonist [98]. TIGIT has emerged as an additional checkpoint inhibitor for novel combinatorial therapy with IL-15 [99].

Additional anti-cancer effects were also observed when IL-15 was delivered together with anti-CD40 agonist antibodies in TRAMP-C2 prostatic tumor-bearing mice [67,100]. The combination therapy induced a 10-fold increase in the number of TRAMP-C2-specific CD8^+^ T cells, thus resulting in tumor growth control. Mechanistically, the stimulation via CD40 rescues CD4^+^ T cell helper activity, promoting the generation of tumor-specific CD8^+^ T cells [101].

Due to the stimulatory effects on NK cells, IL-15 agents have been used in combination with anti-cancer monoclonal antibodies, resulting in increased antibody-dependent cell cytotoxicity (ADCC) and anti-tumor efficacy. Positive results were reported for the combination of *sch* rhIL-15 with cetuximab in breast cancer models [102,103], with rituximab against chronic lymphocytic leukemia (CLL) [104] and EL4 lymphoma transfected with human CD20 [105] and with alemtuzumab in a xenograft model of adult T cell lymphoma (ATL). Preclinical studies also supported the use of N-803 in combination with anti-CD20 monoclonal antibodies, to enhance NK cell function and ADCC against B cell lymphoma [85,106]. Advances also include the use of N-803 together with bi/tri-specific antibodies (BiKE and TriKE), to enhance the survival and expansion of NK cells in vivo. BiKE and TriKE work by creating an immunological synapse between tumor and NK cells. These molecules contain single-chain variable fragments (scFvs) against both CD16 activating receptors on NK cells and tumor associated antigen(s), such as CD19, CD20 and CD33, connected by human IL-15 [107,108,109]. Other receptors such as NKG2D and 2B4, when triggered in concert, have been shown to activate NK cells to a similar degree as engagement of CD16 alone. Fusion proteins comprising RLI have also been developed to further enhance anti-tumor effectiveness. A fusion protein of RLI with anti-GD2 antibody that targets the widely expressed tumor-associated antigen disialoganglioside displayed strong antitumor activities in the subcutaneous EL4 and metastatic NXS2 mouse models via enhanced ADCC [110]. Additionally, trifunctional fusion proteins composed of a tumor-specific recombinant antibody, RLI, and the extracellular region of co-stimulation molecules 4-1BBL, OX40L or glucocorticoid-induced TNF receptor ligand (GITRL), were effective in reducing lung metastasis in the B16-FAP melanoma mouse model, through the induction of T cell proliferation and cytotoxicity, and the secretion of IFN-γ [111,112].

### 5.2. Combination of IL-15 with Chemotherapy and Radiation

Combination regimens of IL-15 with chemotherapy or radiation have also shown efficacy in mice. Both chemotherapy and radiation directly reduce tumor burden and support the development of tumor-specific immune responses through the release of tumor antigens from necrotic cells and by depleting immunosuppressive populations within the tumor microenvironment. Indeed, IL-15 administration potentiates the anti-cancer efficacy of cyclophosphamide [113,114], cisplatin [115], gemcitabine [116] and radiation [117]. Combination therapies of hetIL-15 with chemotherapy and surgical resection of primary tumors are currently under evaluation.

### 5.3. Combination of IL-15 with Adoptive Cell Therapy

Adoptive cell transfer (ACT) therapy is a promising strategy to fight cancer and consists of the administration of patient-derived TILs, T cells specific for tumor epitopes, or chimeric antigen receptor engineered T (CAR-T) or CAR-NK cells. The first anti-CD19 CAR-T cell therapy was approved by the FDA for hematological malignancies in 2017 [118]. ACT therapy effectiveness has been linked to both the phenotype of the transferred cells and their in vivo persistence. Beneficial effects of IL-15 in ACT immunotherapy protocols have been reported in animal models and are being explored in the clinic. IL-15 can be used for the ex vivo generation and expansion of tumor-specific lymphocytes, as well as for the in vivo support of the transferred cells.

Ex vivo pre-culture with IL-15 resulted in the generation of anti-tumor CD8^+^ T cells with the central memory phenotype. In comparison to IL-2, T cell clones generated in the presence of IL-15 displayed higher proliferative capability and cytokine secretion potential and were effective in causing tumor regression upon transfer in mice [61]. Similarly, in a macaque model, IL-15-stimulated Cytomegalovirus-specific CD8 autologous clones were characterized by a central memory rather than terminally differentiated effector phenotype and persisted in vivo for longer periods of time [119]. IL-15 was also reported to promote the in vitro expansion of human T cells with stem cell-like features [120]. These cells are multipotent, self-renewing, and capable of generating potent tumor-specific effector T cells upon transfer in vivo [121]. Indeed, CAR-T cells expanded ex vivo with IL-15 were characterized by a less-differentiated phenotype, with reduced expression of exhaustion and pro-apoptotic molecules, and by an improved mitochondrial metabolism [122].

The in vivo persistence of adoptively transferred cells benefits from lymphodepleting pre-conditioning of the host before cell infusion. It has been shown that hetIL-15 administration improved the outcome of ACT therapy in the absence of lymphodepletion in a B16 melanoma mouse model [72], establishing an efficient protocol of ACT. Treatment with hetIL-15 resulted in tumor infiltration and persistence of both adoptively transferred gp100-specific Pmel-1 cells and endogenous CD8^+^ T cells. Importantly, hetIL-15 treatment led to preferential tumor enrichment and increased cytotoxic ability of tumor-infiltrating Pmel-1 cells, resulting in improved tumor control and survival. Thus, hetIL-15 administration improves the outcome of ACT in immunocompetent hosts, a finding with significant implications for improving future cell-based cancer therapy strategies. These results may also provide methods to extend ACT to patients that cannot be treated with lymphodepleting regimens. In a human CD19 CAR-T study, it was reported that high IL-15 plasma levels at the time of CAR-T infusion were associated with the effectiveness of this treatment [123].

In an effort to develop new generation CAR-T cell-based therapy, the sequence encoding for a membrane-bound IL-15 (mbIL-15) fusion protein has been incorporated into the CAR-expressing lentiviral vectors. CD19 CAR-T cells co-expressing mbIL-15 were long-lived, possessed the stem-cell like CD45RO^neg^CCR7^+^ phenotype, and mediated the rejection of CD19^+^ leukemia in mice [124]. The employment of tethered IL-15 in combination with CAR-T cells shows promise for interventions against solid tumors. Indeed, coexpression of mbIL-15 in IL-13Rα2 CAR-T cells provided benefits in a mouse model of glioblastoma [125]. New generation CAR-T cells co-expressing mbIL-15 displayed high levels of Bcl-2, low levels of PD-1, and were characterized by enhanced tumor localization and effector functions. Their anti-tumor effect was also associated with the modification of the tumor microenvironment, with enhanced NK cell activation and a reduced number of M2 macrophages [126]. Such therapies may raise concerns regarding uncontrolled T cell proliferation as a consequence of the continuous exposure to IL-15. To overcome this issue, a new strategy consists of the incorporation of the suicide gene inducible caspase-9 (iC9) into CD19 CAR-T/mbIL-15 cells. These cells displayed great proliferative capability both in vitro and in vivo, increased survival, a reduced exhaustion phenotype, and improved anti-tumor effects. Additionally, these cells could be safely eliminated through exogenous stimulation of the iC9 gene [127]. The co-expression of mbIL-15 has also proved advantageous in CD19 CAR-NK against hematological malignancies [128,129].

Overall, both pre-treatment with IL-15 and in vivo combination of IL-15 with CAR-T, CAR-NK and TILs provides advantages to treat cancers (for reviews, see also refs [130,131,132].

### 5.4. γ-Chain Family of Cytokines

The anti-cancer effects of IL-15 are based on the activation of CD8^+^ T and NK cells. Other cytokines belonging to the γ-chain family exert similar effects and can be used for cancer treatment, either alone or in combination with IL-15. IL-2 is the prototypical cytokine employed as a cancer immunotherapeutic agent. It was approved for clinical use in 1992 for the treatment of certain types of human malignancies, such as melanoma, renal carcinoma, and non-Hodgkin’s lymphoma. IL-2 stimulates NK and terminally-differentiated effector CD8^+^ T cells with high cytolytic activity [133,134]. IL-2, as well as other γ-chain cytokines, have also been reported to support ACT in the B16 mouse melanoma model [135]. However, the role of IL-2 in activation induced cell death (AICD), and in promoting the accumulation of Tregs, especially at low doses [136,137], partially counteracts its anti-tumor effectiveness. Additionally, the use of high-dose recombinant IL-2 is currently limited by the severe toxic effects associated with the therapy such as capillary leak syndrome, hypotension, and renal insufficiency [138]. These detrimental factors have suggested a need for cytokine therapies possessing the immunostimulatory effects of IL-2, but with fewer adverse effects. Among these, the use of IL-2/anti-IL-2R antibody complexes showed promising results in mouse tumor models. The antibody employed in the complexes stabilize the cytokine and favors the specific binding to cells expressing the β/γ receptor, limiting the activity of IL-2 to CD8^+^ T and NK cells, but not IL-2Rα^+^ Tregs [139,140,141,142,143].

## 6. IL-15 in Clinical Trials for Cancer Immunotherapy

The success of IL-15-based immunotherapies in preclinical studies has led the development of several registered clinical trials, involving administration of IL-15 alone or with other immunotherapy. Several delivery routes, including IV, SC and CIV, are currently being tested. An overview is presented in Table 1.

### 6.1. sch rhIL-15

The first human phase I trial consisted of the administration of *sch* rhIL-15 via IV route for 12 consecutive days in patients with metastatic cancers [144]. Toxicity at the doses of 3 and 1 µg/kg/day were observed, while all nine patients treated with a dose of 0.03 µg/kg/dose, identified as the maximum tolerated dose (MTD), were able to complete the treatment without dose-limiting toxicity. At higher doses, the toxicities observed were grade 3 hypotension and thrombocytopenia, as well as persistent elevated aminotransferase levels. Additional adverse effects in patient with 3 µg/kg/day include fever and chills, mostly related to the markedly elevated levels of circulating inflammatory cytokines IL-6 and IFN-γ. IV treatment with *sch* rhIL-15 resulted in a rapid extravasation of NK and effector T cells from peripheral blood within minutes after IL-15 delivery, followed by increased proliferation and gradual accumulation of circulating NK, γδ T and memory CD8^+^ T cells [144]. As monotherapy, *sch* rhIL-15 was also administered via SC route at 0.25, 0.5, 1, 2 and 3 µg/kg/day for five consecutive days for two weeks [145] and by CIV at 0.125, 0.25, 0.5, 1, 2 and 4 µg/kg/day for 10 consecutive days [146]. Upon SC delivery, serious adverse events included grade 2 pancreatitis and grade 3 cardiac chest pain, associated with hypotension and elevated troponin. The treatment induced a great expansion of circulating NK cells, especially in the CD56^brigh^ subset, and to a lesser extent of CD8^+^ T cells at the identified MTD of 2 µg/kg/dose [145]. The major challenges in both IV and SC trials were the short in vivo half-life of *sch* rhIL-15 and the risk of immunogenicity toward the non-glycosylated cytokine. Upon CIV administration, the highest tested doses induced dose-limiting toxicity and MTD was 2 µg/kg/dose. CIV provided the most effective way to deliver *sch* rhIL-15. The IL-15 C_max_ was at 48 h, followed by a decline to 8% of maximum level by day 8–10. Similarly to IV administration, at the beginning of CIV treatment, extravasation of NK and CD8^+^ memory T cells occurred, followed by a gradual increase and peak accumulation of circulating lymphocytes shortly after termination of infusion. Patients in the 2 μg/kg/day group showed a remarkable 358-fold increase in circulating CD56^bright^ NK cells, and 38-fold increase in total NK cells. The number of circulating CD8^+^ T cells increased by 5.8-fold [146]. In all three trials, the best response observed was stable disease.

Combination therapy of *sch* rhIL-15 with other agents is also being explored in the clinic. In two trials, *sch* rhIL-15 was delivered by IV and SC routes [147] after lymphodepleting regimen and haploidentical NK cell infusion in patients with acute myeloid leukemia, resulting in 35% remission. Several phase I trials have been initiated, including *sch* rhIL-15 with nivolumab (a-PD-1) and ipilimumab (a-CTLA4) in refractory cancers (NCT03388632), with avelumab (a-PD-L1; NCT03905135) in T cell malignancies, with obinutuzumab (a-CD20; NCT03759184) in CLL; and with alemtuzumab (a-CD52; NCT02689453) in ATL.

### 6.2. hetIL-15 (NIZ985)

A phase I clinical trial (NCT02452268) has been conducted at the NIH Clinical Center having the following primary objectives: (1) to characterize the safety and tolerability of hetIL-15 in adults with metastatic cancers; (2) to identify the MTD and/or recommended dose for expansion (MTD/RDE) of hetIL-15 (NIZ985). hetIL-15 was delivered subcutaneously three times per week at doses of 0.25, 0.5, 1, 2 or 4 μg/kg for the first two weeks of each 28-day cycle (manuscript in preparation). Beginning with the first dose level, patients developed erythematous injection site reactions characterized by significant perivascular T cell infiltration, dermal macrophage infiltration, and accumulation of CD56^+^ cells, likely NK cells, at the dermal–epidermal junction. No MTD was reached and stable disease was the best response. NIZ985 treatment was associated with increases in several cytokines, including IFN-γ, IL-18, CXCL10, and TNF-β, plus significant induction of cytotoxic lymphocyte proliferation (including NK and CD8^+^ T cells). A phase I/Ib multicenter study of hetIL-15 (NIZ985) in combination with PDR001 (Anti-PD-1) in adults with metastatic cancers has also being initiated with the objectives: (1) to characterize the safety and tolerability of hetIL-15 alone and in combination with PDR001; and (2) to identify the MTD/RDE of hetIL-15 alone and in combination with PDR001. The clinical study has been expanded to a multicenter study (NCT02452268). An additional study combining hetIL-15 delivered via SC route with spartalizumab has been initiated in Japan in patients with advanced solid tumors and lymphoma who have progressed after obtaining a previous response to checkpoint inhibitor therapy (NCT04261439).

### 6.3. N-803 (Anktiva, Formerly ALT-803)

N-803 was administered to patients with hematological malignancies once weekly for four weeks, at doses of 1, 3, 6 and 10 µg/kg/dose via IV route and at doses of 6 and 10 µg/kg/dose via SC route [148], and to patients with advanced solid tumors for four consecutive weeks, every six weeks at a dose of 20 µg/kg/dose, via IV or SC routes, respectively [149]. No dose-limiting toxicities were observed. IV injections were associated with fatigue and nausea, related to the plasma spike in IL-6 and IFN-γ [149]. SC administration resulted in large, painful erythematous plaques at the injection site, associated with infiltration of CD56^+^NKp46^−^ γδ T cells [148]. Pharmacokinetic analysis showed prolonged serum concentrations with a peak between 2 h and 6 h following SC, but not IV, injection. N-803 stimulated the proliferation and activation of circulating NK cells and CD8^+^ T cells, without affecting Tregs [148]. In patients with hematological malignancies, responses were observed in 19% of patients, with three patients presenting stable disease, one with partial response, and one with complete remission lasting longer than seven months [148]. No responses were observed in patients with solid tumors [149]. Low-titer antibodies in one treated patient was reported [148]. A phase I trials of N-803 delivered subcutaneously in combination with nivolumab in patients with metastatic non-small cell lung cancer has also been conducted [150]. The treatment was well-tolerated, with the most common adverse effects being injection site reactions, flu-like symptoms, and fatigue. A grade 3 myocardial infarction occurred in one patient. MTD was not reached, and the recommended dose for phase II was 20 μg/kg N-803 given once per week subcutaneously, in combination with 240 mg IV nivolumab every two weeks. Changes in peripheral blood mainly affected NK cells (three-fold increase) and were more modest in CD8^+^ T cells. Stable disease and partial responses were observed in 10 and 6 of 21 patients, respectively. Antibodies against N-803 were found in 33% of the treated patients [150]. More combination trials are ongoing with rituximab (NCT02384954) and pembrolizumab/nivolumab/atezolizumab (NCT03228667).

## 7. Conclusions

IL-15 is a promising agent for cancer immunotherapy. IL-15 functions by stimulating effector immune cells capable of killing cancer cells. Several preclinical studies have shown that IL-15 promotes tumor infiltration of both lymphocytes and dendritic cells, through the induction of several cytokines, and supports the proliferation, survival, and cytotoxic activity of both CD8^+^ T and NK cells. Despite the success of *sch* rhIL-15 in preclinical studies, its use in clinics is limited by the short half-life in vivo and potential toxicity associated with high-dose delivery. IL-15 is produced in the body as a heterodimeric cytokine comprising the IL-15 and IL-15Rα chains, named hetIL-15. Both hetIL-15 and variants of IL-15:IL-15Rα complexes (N-803 and RLI) showed improved pharmacokinetics and anti-tumor activity in animal models and in humans. The hetIL-15 molecule has certain advantages compared to the other versions of IL-15, currently in clinical trials, which are: (i) single-chain IL-15 produced in *E. coli* [53] has a short plasma half-life [144] and is immunogenic in humans; and (ii) N-803 has altered structure and glycosylation and may be immunogenic in humans [148,150]. In contrast, hetIL-15 represents the endogenous molecule circulating in the blood, and no antibodies against it have been detected so far after administration in humans. The available data suggest that N-803 and hetIL-15 have comparable biological activities.

Current challenges aim to identify optimal dosing schemes and routes of administration to maximize anti-tumor effects and minimize toxicity; and to select optimal combinations with other anti-cancer agents and immunotherapies.

## Figures and Tables

**Table 1:** Clinical trials with IL-15.

IL-15 Agents	Combination Immunotherapy	Route of IL-15 Delivery	Dose Tested or MTD ^1^	Application	Best Clinical Response	NCT/ Reference
*sch* rhIL-15	-	IV	0.3 µg/kg/dose daily for 12 days	Malignant melanoma and renal cell carcinoma	SD	[144]
*sch* rhIL-15	-	SC	2 µg/kg/dose 5 days/week for 2 weeks	Advanced solid tumors	SD	[145]
*sch* rhIL-15	-	CIV	2 µg/kg/dose for 10 days	Metastatic tumors	SD	[146]
*sch* rhIL-15	Lympho-depleting regimen and haploidentical NK cell infusion	IV/SC	0.3 µg/kg/dose daily for 12 days IV; 2 µg/kg/dose for 10 days SC	Acute myeloid leukemia	35% remission	[147]
*sch* rhIL-15	Nivolumab and Ipilimumab	SC	Daily on day 1–8 and 22–29	Refractory cancers	Active/ recruiting	NCT03388632
*sch* rhIL-15	Avelumab	CIV	1, 2, 3 and 4 µg/kg/day for 5 days (max 6 cycles)	Refractory T cell malignancies	Active/ recruiting	NCT03905135
*sch* rhIL-15	Obinutuzumab	CIV	0.5, 1 and 2 µg/kg/day for 5 days (max 6 cycles)	CLL	Active/ recruiting	NCT03759184
*sch* rhIL-15	Alemtuzumab	SC	0.5–2 µg/kg/dose 5 days/week for 2 weeks	ATL	Active/ recruiting	NCT02689453
hetIL-15 (NIZ985)	-	SC	0.25, 0.5, 1, 2 or 4 μg/kg 3 times/week for 2 weeks	Metastatic solid tumors	Active	NCT02452268
hetIL-15 (NIZ985)	PDR001 /Spartalizumab	SC	0.25, 0.5, 1, 2 or 4 μg/kg 3 times/week for 2 weeks	Metastatic solid tumors	Active	NCT02452268
hetIL-15 (NIZ985)	Spartalizumab	SC	2 or 4 μg/kg weekly	Advanced solid tumors and lymphoma (checkpoint inhibitors relapsed)	Active	NCT04261439
N-803	-	IV/SC	10 µg/kg/dose IV for 10 days or SC weekly for 4 weeks	Hematological malignancies	1 CR, 1 PR, 3 SD	[148]
N-803	-	IV/SC	20 µg/kg/dose weekly for 4 weeks, every 6 weeks	Solid tumors	No response	[149]
N-803	Nivolumab	SC	20 µg/kg/dose + 240 mg nivolumab iv every 2 weeks	Non-small cell lung carcinoma	6 PR, 10 SD	[150]
N-803	Rituximab	IV/SC	Weekly for 4 weeks	B-cell non-Hodgkin’s lymphoma	Active	NCT02384954
N-803	Pembrolizumab/ Nivolumab/ Atezolizumab	SC	15 µg/kg/dose every 3 weeks	Advanced cancers	Active/ recruiting	NCT03228667
N-803	Standard-of-care chemotherapy/ aldoxorubicin HCl/ PD-L1 t-haNK	SC	15 µg/kg/dose every 3 weeks	Pancreatic cancer	Active/ recruiting	NCT04390399

^1^ MTD, maximum tolerated dose; NCT, National Clinical Trial; IV, intravenous; SC, subcutaneous; CIV, continuous intravenous infusion; SD, stable disease; PR, partial response; CR, complete response.
